# Reconsidering Association Testing Methods Using Single-Variant Test Statistics as Alternatives to Pooling Tests for Sequence Data with Rare Variants

**DOI:** 10.1371/journal.pone.0030238

**Published:** 2012-02-17

**Authors:** Daniel D. Kinnamon, Ray E. Hershberger, Eden R. Martin

**Affiliations:** 1 Dr. John T. Macdonald Foundation Department of Human Genetics, Miller School of Medicine, University of Miami, Miami, Florida, United States of America; 2 Cardiovascular Division, Miller School of Medicine, University of Miami, Miami, Florida, United States of America; University of Bristol, United Kingdom

## Abstract

Association tests that pool minor alleles into a measure of burden at a locus have been proposed for case-control studies using sequence data containing rare variants. However, such pooling tests are not robust to the inclusion of neutral and protective variants, which can mask the association signal from risk variants. Early studies proposing pooling tests dismissed methods for locus-wide inference using nonnegative single-variant test statistics based on unrealistic comparisons. However, such methods are robust to the inclusion of neutral and protective variants and therefore may be more useful than previously appreciated. In fact, some recently proposed methods derived within different frameworks are equivalent to performing inference on weighted sums of squared single-variant score statistics. In this study, we compared two existing methods for locus-wide inference using nonnegative single-variant test statistics to two widely cited pooling tests under more realistic conditions. We established analytic results for a simple model with one rare risk and one rare neutral variant, which demonstrated that pooling tests were less powerful than even Bonferroni-corrected single-variant tests in most realistic situations. We also performed simulations using variants with realistic minor allele frequency and linkage disequilibrium spectra, disease models with multiple rare risk variants and extensive neutral variation, and varying rates of missing genotypes. In all scenarios considered, existing methods using nonnegative single-variant test statistics had power comparable to or greater than two widely cited pooling tests. Moreover, in disease models with only rare risk variants, an existing method based on the maximum single-variant Cochran-Armitage trend chi-square statistic in the locus had power comparable to or greater than another existing method closely related to some recently proposed methods. We conclude that efficient locus-wide inference using single-variant test statistics should be reconsidered as a useful framework for devising powerful association tests in sequence data with rare variants.

## Introduction

The advent of high-throughput sequencing technologies is providing an unprecedented opportunity to examine the association of both common and rare variation with disease on an exome-wide, and soon genome-wide, scale. In this study, we consider the problem of using sequence data from a case-control sample to perform a test for association between a disease and a locus, which we define as a region of contiguous sequence including many variants (i.e., polymorphic sequence positions). These variants may be either common or rare; those with minor allele frequencies (MAFs) <2–3% [1] are termed rare variants, and those with higher MAFs are termed common variants. Under the null hypothesis of no association with the locus, all multi-variant genotypes have the same disease risk. This implies that cases and controls have equal multi-variant genotype frequencies, and therefore single-variant genotype frequencies, at the locus under the null hypothesis. Under the alternative hypothesis, multi-variant genotypes have disease risks depending on one or more variants in the locus, meaning that multi-variant genotype frequencies differ between cases and controls at the locus.

To mitigate the power loss due to allelic heterogeneity [1]–[4] and high dimensionality in this scenario, conventional wisdom suggests that pooling minor alleles at rare variants into a measure of burden at a locus will be necessary to detect associations [4]–[6]. Tests based on either collapsing rare variants in a locus into a single indicator of the presence of any minor alleles [5], [7] or summing weighted minor allele counts over rare, and sometimes also common, variants in a locus [6], [8]–[10] have been proposed as alternatives to single-variant tests. We subsequently refer to techniques involving collapsing or summing as pooling tests. Two of the earliest proposals, the Combined Multivariate and Collapsing (CMC) method [5] for collapsing and the Weighted Sum Statistic (WSS) method [6] for summing, are commonly used as benchmarks for novel methods in the rare variant association testing literature [8]–[13] based on results suggesting that these techniques were superior to locus-wide inference using single-variant test statistics.

The power of pooling tests will depend on the linkage disequilibrium (LD) patterns in sequence data. Simulations using a coalescent approximation to a neutral two-locus Wright-Fisher infinite allele model have shown that a substantial proportion of the pairwise LD between biallelic variants can be expected to be negative (i.e., *D*<0), even at very high levels of recombination [14]. Most importantly for sequence data, negative pairwise LD values become more likely when including variants with relatively rare minor alleles [14]. To the extent that there is negative LD between neutral and risk variants within a locus, higher MAFs at a small number of risk variants in cases will be accompanied by higher MAFs at a larger number of neutral variants in controls. In this situation, case-control differences in the MAFs at individual risk variants may actually be masked by those at a large number of neutral variants in locus-wide summaries based on collapsing or summing minor alleles over all variants at a locus. Masking, in turn, reduces the power of pooling tests by obscuring locus-wide case-control differences. Such masking and power loss will be exacerbated by the inclusion of protective variants.

Because pooling tests lose power when neutral and protective variants are included, one sensible approach is to try to exclude such variants a priori by filtering on annotation and functional predictions. However, making such exclusions with high sensitivity and specificity will be difficult, particularly in non-coding regions for which little information is available. Even in coding regions, functional predictions may lead researchers astray. For example, recent studies have implicated synonymous variants in altering the function of protein products [15] and causing disease [16]. Thus, methods for locus-wide inference that are inherently robust to the inclusion of neutral and protective variants are desirable.

Existing methods for locus-wide inference using nonnegative single-variant test statistics, such as performing joint inference based on the maximum Cochran-Armitage trend chi-square statistic in the locus, are inherently robust to the inclusion of neutral and protective variants. This robustness arises from the fact that the locus-wide inference depends on only the magnitude of the deviation from the null hypothesis at each variant and not the direction. Joint inference can be performed efficiently by using permutation, which, by simulating draws from the joint null distribution of the single-variant test statistics, avoids conservative approximations (e.g., the Bonferroni correction) and accounts for LD-induced correlations between test statistics [17]. Existing methods that perform efficient locus-wide inference on nonnegative single-variant test statistics using permutation can therefore combine information across variants in a locus without masking. Such methods may also be able to extract additional association signal from neutral variants in negative LD with risk variants as well as protective variants. Finally, such methods allow use of all available data when genotypes are missing completely at random in the sense of Little and Rubin [18], which is not necessarily true of pooling tests.

Nonetheless, many novel methods have not departed from the pooling test framework but rather attempted to devise improved weighting schemes and adaptive thresholds that reduce the influence of neutral and protective variants [8]–[10], [13]. Despite deriving their approaches within fundamentally different frameworks, some of the newest methods that are robust to the inclusion of neutral and protective variants have actually arrived at procedures equivalent to performing locus-wide inference using nonnegative single-variant test statistics. For example, the Sequence Kernel Association Test (SKAT) [13] and the C-alpha test [12] base inference on weighted and unweighted sums of squared single-variant score statistics, respectively [13]. Under an additive genetic model without covariates and with variant weights equal to the inverse of the estimated null variance of each single-variant score statistic, the SKAT statistic is simply the sum of single-variant Cochran-Armitage trend chi-square statistics. Sums of nonnegative single-variant test statistics have previously been recommended as powerful methods for joint inference over multiple variants in candidate gene association studies [19] and genome-wide association studies (GWAS) [20], [21]. The equivalence of new developments to existing methods for efficient locus-wide inference using nonnegative single-variant test statistics argues for broader investigation of other existing methods falling within this framework, such as permutation inference on the maximum single-variant Cochran-Armitage trend chi-square statistic in the locus.

In this study, we compared two existing methods for locus-wide inference using nonnegative single-variant test statistics to the two originally proposed pooling tests [5], [6] under more realistic conditions. We began by examining the characteristics of variants appearing in actual candidate gene sequence data. We then illustrated with analytic power calculations using a simple model how pooling tests may have lower power than even Bonferroni-corrected single-variant tests in the presence of neutral variants. We finally extended the basic conclusions of this simple model to more complex situations with allelic heterogeneity, extensive neutral variation, and randomly missing genotype data using simulations based on variants with realistic MAF and LD spectra. In these simulations, we compared the locus-wide type I error and power of a Bonferroni-corrected test based on the maximum single-variant Cochran-Armitage trend chi-square statistic, efficient permutation tests based on the maximum or sum of single-variant Cochran-Armitage trend chi-square statistics, the CMC test, and the WSS test. We found that methods for efficient locus-wide inference using nonnegative single-variant test statistics performed as well as, and often better than, the CMC and WSS tests under a variety of scenarios. For disease models with only rare risk variants, we observed that the permutation test based on the maximum of single-variant Cochran-Armitage trend chi-square statistics had power comparable to or greater than the permutation test based on the sum, which we show is closely related to the SKAT and C-alpha test. We conclude that efficient locus-wide inference using nonnegative single-variant test statistics should be reconsidered as a useful framework for devising powerful association tests in sequence data with rare variants.

## Methods

### Characteristics of Variants in Actual Sequence Data

To ground our interpretation of analytic power approximations in actual data and provide a basis for evaluating the realism of our simulated data, we estimated the MAF and LD distributions of variants in resequencing data from six genes (*CSRP3*, *LDB3*, *MYH7*, *SCN5A*, *TCAP*, and *TNNT2*) previously obtained for a study of candidate genes for dilated cardiomyopathy [22]. These data should provide a useful snapshot of sequence-level variation within protein-coding genes of a wide range of sizes (3 kb–103 kb), numbers of exons (2–40), and chromosomal locations (1q32, 3p21, 10q22.3–23.2, 11p15.1, 14q12, and 17q12). Bi-directional Sanger sequencing of all coding sequence, at least 50 bp into 5′-/3′-UTRs, and at least 40 bp into all introns was performed by SeattleSNPs under contract to the National Heart, Lung, and Blood Institute resequencing service. We used data from 184 unrelated controls of European descent from the Coriell database with variant call rates ≥80% after removal of low-quality variants called in <80% of individuals in the study. We considered biallelic variants with no evidence of deviation from Hardy-Weinberg equilibrium (HWE) (Monte Carlo exact HWE *P*≥0.001), including both SNPs and small insertions/deletions, in calculating the MAF and LD distributions. Pairwise LD measured by the correlation coefficient between major alleles, *r*, was calculated only between variants within the same gene under the assumption of HWE using the method of Weir and Cockerham [23] as implemented in PROC ALLELE, SAS/GENETICS, Version 9.2 (SAS Institute Inc., Cary, NC). This correlation coefficient is the same as the correlation coefficient between minor alleles for a biallelic variant.

### Impact of Neutral Variation in a Simple Model

We began by examining the relative performance of pooling tests and locus-wide inference using nonnegative single-variant test statistics in a simple model of a locus comprising one rare risk variant and one rare neutral variant. Specifically, we compared the locus-wide analytic power of collapsing and summing to that of performing Bonferroni-corrected inference on the maximum single-variant Cochran-Armitage trend chi-square statistic in the locus (the BC-CA test) under our model at varying levels of LD. The Cochran-Armitage test for a single variant is well-known in the field of genetics [24]–[26]. It possesses some desirable properties, including robustness to departures from HWE [24] and ease of calculation, that make it widely applicable. Suppose that we independently sample *R* cases and *S* controls, and let *N* = *R*+*S*. It will be convenient for subsequent exposition to present the test statistic *T* as a generalization of the form presented in Freidlin et al. [26] to multi-variant genotypes:
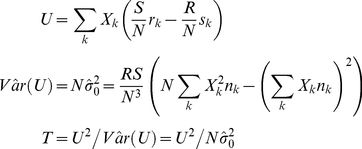
(1)In (1), *X_k_* is a score corresponding to the *k* ^th^ single- or multi-variant genotype, denoted **G_k_** = [*G_1k_*,…,*G_vk_*], comprising *v* biallelic variants indexed by *j*. We also let *r_k_* be the number of cases with genotype **G_k_**, *s_k_* be the number of controls with genotype **G_k_**, and *n_k_ = r_k_+s_k_*. The single-variant genotype at biallelic variant *j* in multi-variant genotype **G_k_** is coded as *G_jk_* = 0, 1, or 2 minor alleles. Thus, multi-variant genotype *k* denotes one unique combination of single-variant genotypes (e.g., **G_k_** = [0,0,1,2] for *v* = 4 and one particular *k*) in the set of all *w* = 3*^v^* possible combinations at each of the *v* variants (i.e., *k* = 1,2,…, 3*^v^*-1,3*^v^*). If we consider a single variant and use scores equaling the number of minor alleles at that variant, we have 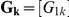, *k* = 1, 2, or 3, and 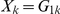, so *T* in (1) reduces to the single-variant Cochran-Armitage trend chi-square statistic.

A simple model of a locus comprising one rare neutral (*j* = 1) and one rare risk (*j* = 2) biallelic variant with the same MAF (*p*) was used. In this model, frequencies of haplotypes [*h_1_*,*h_2_*] for a given level of LD (*D*) were calculated as (1-*p*)^2^ *+D* for haplotype [0,0], (1-*p*)*p*-*D* for haplotypes [0,1] and [1,0], and *p* ^2^ *+D* for haplotype [1,1], where 1 denotes the minor allele and 0 the major allele at either variant. Population frequencies of multi-variant genotypes **G_k_** = [*G_1k_*,*G_2k_*], denoted by *p_k_*, were then determined under HWE. A multiplicative risk model, 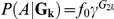, was used, with *A* or *C* denoting affection status (affected case or control), *f_0_* denoting the penetrance for the variant 2 major allele homozygote, and *γ* denoting the relative risk for an additional minor allele at variant 2. The conditional frequencies of each **G_k_** in cases (*p_k|A_*) and controls (*p_k|C_*) were determined based on this risk model and population multi-variant genotype frequencies using Bayes' rule:
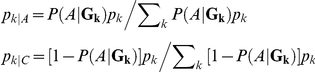
Under the generalization in (1) and the assumed sampling model, 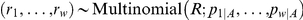 and 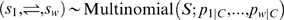. The null hypothesis that no variant in the locus influences disease risk implies that 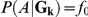 and 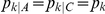 for all *k*. Under the alternative hypothesis, at least one variant in the locus influences disease risk, implying that 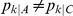 for some *k*.

Freidlin et al. [26] provide formulas for the variance under this null hypothesis as well as the expectation and variance under this alternative hypothesis for the statistic *U* in (1) with arbitrary scores *X_k_* when *v* = 1. These formulas immediately generalize to multinomial genotype distributions with more than 3 possible genotypes (*w*>3):
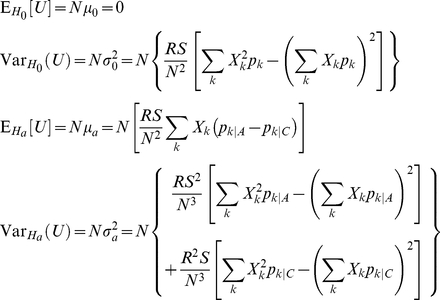
Under the null hypothesis, the asymptotic distribution of *U* is 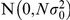, and 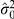 defined in (1) converges in probability to 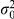 [26]. Thus, *T* has the same asymptotic 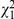 null distribution as 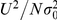 by Slutsky's Theorem because 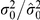 converges in probability to 1 [27]. Under the alternative hypothesis, the asymptotic distribution of *U* is 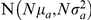 and 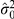 converges in probability to [26]:
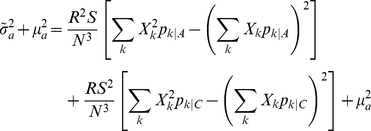
Thus, the asymptotic power function for any two-sided test based on *T* with type I error rate *α* is:
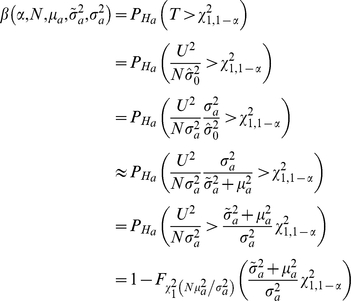
 where 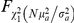 refers to the noncentral *χ* ^2^ CDF with 1 degree of freedom and noncentrality parameter 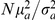 [28].

We show in Appendix A (Text S1) that particular choices of scores *X_k_* in the statistic *T* yield a single-variant Cochran-Armitage trend test, a collapsing test, and a summing test. This correspondence allows us to use the generalized formulas presented above to approximate the locus-wide asymptotic power of each approach to association testing in our model. The single-variant trend test uses 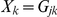 for variant *j*; collapsing defines 
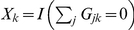
, where *I*(*E*) = 1 if *E* is true and 0 otherwise; and summing defines 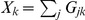. A slight modification is required to approximate the locus-wide power of the BC-CA test in the presence of LD because calculating the joint distribution of the single-variant test statistics is analytically intractable. In Appendix B (Text S1), we establish that a lower bound for the locus-wide power function of the BC-CA test is the power of the Bonferroni-corrected Cochran-Armitage trend test for the risk variant alone.

We examined power as a function of 
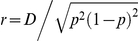
 for *f*_0_ = 0.05 and two types of rare variant pairs (*p* = 0.005 and *γ* = 3; *p* = 0.01 and *γ* = 2). Balanced case-control samples with total sizes of *N* = 500, 1,000, and 2,000 were considered. Values of *r* were chosen by taking 100 evenly spaced increments of *D* starting from *D*_min_ = −*p* ^2^ and ending at *D*_max_ = *p*(1-*p*).

### Monte Carlo Simulations

We used Monte Carlo simulations to extend the conclusions drawn from the two-variant locus model to a larger locus with heterogeneous risk alleles, extensive neutral variation, realistic LD patterns, and randomly missing genotype data. We begin by outlining the major components of the simulation procedure and subsequently provide more detailed exposition for each component. One thousand populations of haplotypes at a 100 kb locus with realistic MAF and LD spectra were first generated based on a neutral coalescent model. Using the same 1,000 haplotype populations, a separate simulation was then conducted for each combination of user-specified risk variant parameters and sample size. Within each simulation:

1. The disease model for each haplotype population was generated by randomly selecting risk variants for inclusion based on user-specified parameters;
2. A case-control sample was drawn from each haplotype population according to the disease model and a user-specified sample size;
3. Data sets with randomly missing genotypes were generated from each sample for each user-specified call rate; and
4. All association testing techniques were applied to each data set.

Type I error and power for each technique were estimated for balanced case-control samples of total sizes *N* = 500, 1,000, and 2,000. The disease risk for the multi-variant genotype with no minor alleles at any risk variant was 5% for all simulations. For power, 50 risk variants with independent effects were randomly selected in each haplotype population under three different disease models:

1. Multiple rare risk variants (MAF<0.005; odds ratio (OR) = 3);
2. Multiple rare risk variants (MAF<0.01; OR = 2);
3. Combinations of multiple rare risk variants (MAF<0.01; OR = 2), low-frequency risk variants (0.01≤MAF<0.05; OR = 1.5), and common risk variants (0.05≤MAF<0.10; OR = 1.2).

The first two models represent situations in which pooling tests are expected to perform best, and the third model is included to consider cases where both common and rare variants might contribute to disease susceptibility. We chose a number risk variants that represented ∼5% of all variants at the locus in the average haplotype population to reflect a situation in which associations between the locus and disease were driven by heterogeneous risk alleles characterized by a small number of risk variants among a much larger number of neutral variants. Per-position genotype call rates of 100% (complete data), 99.5%, and 95% were simulated. In the following subsections, we provide a detailed description of each component of the simulation procedure.

### Haplotype Populations

One thousand populations of 10,000 haplotypes each were generated at a 100 kb locus, which is representative of a larger human protein coding gene based on recent data (mean size: 27 kb, range: 1 kb–2,400 kb) [29]. Haplotype populations were generated according to a standard neutral coalescent approximation to the Wright-Fisher model with a finite-sites recombination model and an infinite-sites mutation model, which is most accurate when the number of haplotypes sampled is small relative to the number of haplotypes in the population and the recombination rate between adjacent bases is small [30], [31]. We used a per-nucleotide neutral mutation rate of 2.5×10^−8^ estimated assuming an effective diploid population size of *N_e_*≈10,000 [32], a recombination rate of 1×10^−8^ between adjacent nucleotides derived by using the approximation 1 cM≈1 Mb [33], and *N_e_* = 10,000 (i.e., 20,000 haplotypes). We used the program MS [31] and, following the suggestion in the documentation, replaced the standard C random number generator with the well-known and highly robust Mersenne-Twister random number generator [34]. All variants generated by MS are biallelic because it assumes the infinite-sites model of mutation.

### Disease Model

Disease risk for the multi-variant genotype **G_k_** was determined according to a logistic penetrance model of the form:
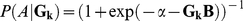
(2)where 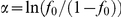, the log odds of the wild-type penetrance for the multi-variant genotype with no minor alleles at any risk variant, and **B** = [*β_1_*,…,*β_v_*]^T^ is the vector of log odds ratios for the haplotype population. The odds ratio 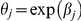 reflects the increase in the odds of disease for each additional minor allele at variant *j*. This model implicitly assumes that (1) each additional minor allele has a multiplicative effect on the odds of disease and (2) this effect at variant position *j* is independent of the effects at other variant positions.

To parameterize this model, we specified the desired number of risk variants as well as a set of risk variant classes indexed by *c*, where class *c* was defined by a half-open MAF range, 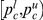, and an associated odds ratio, 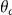. For each haplotype population, the vector of log odds ratios **B** was populated by repeating the following steps until the specified number of risk variants was selected:

1. A variant *j* was randomly selected from among the variants in the haplotype population;
2. If the randomly selected variant *j* had a population MAF in the interval 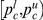 specified for risk variant class *c* and had not already been designated a risk variant, then it was labeled a risk variant and assigned a coefficient 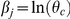.

This procedure effectively randomly samples risk-variant classes from the haplotype population in proportion to the occurrence of each MAF range in the population. All neutral variants had *β_j_* = 0 (*θ_c_* = 1).

### Case-Control Samples

After the vector of log odds ratios **B** was populated for a haplotype population, a case-control sample was generated according to the disease model. To generate a case-control sample, the following procedure was repeated until the user-specified numbers of case and control subjects were selected:

1. Haplotypes were randomly drawn with replacement to form an individual's multi-variant genotype **G_k_**;
2. The disease risk of the individual's multi-variant genotype, *P*(*A*|**G_k_**), was then calculated according to the logistic model in (2);
3. The individual was randomly assigned affection status *A* with probability *P*(*A*|**G_k_**) or *C* with probability 1-*P*(*A*|**G_k_**).

Our method of forming **G_k_** by randomly drawing haplotypes with replacement implicitly assumes random mating in the population.

### Missing Genotypes

For each case-control sample, data sets with different rates of randomly missing genotypes were generated based on user-specified per-base-pair call rates. The observation process over the sequence was modeled as a two-state Markov chain with states “observed” (*O*) or “missing” (*M*) at each position defined by a single base pair. Given a call rate of *λ*<1 per base pair, the number of base pairs that a chain remains in *M* before a genotype is called is distributed Exp(*λ*), assuming a sufficiently long sequence for continuous measurement of base pair position to be reasonable. At the position of this called genotype, the state of the chain changes from *M* to *O* with probability 1. Because genotypes are missing at a rate of 1-*λ* per base pair, the number of base pairs that the chain remains in *O* before a genotype is missing is distributed Exp(1-*λ*), and the chain transitions from *O* to *M* with probability 1 at this position. If we rescale position to [0, 1] by measuring in units of *L* base pairs, where *L* is the total sequence length, the distance the chain remains in *M* is distributed Exp(*Lλ*), and the distance the chain remains in *O* is distributed Exp(*L*(1-*λ*)). It can be shown that the expected proportion of the sequence length that a Markov chain with these transition rates and probabilities spends in *O* is simply the call rate, *λ*. Thus, for each call rate, the following steps were performed in each individual to generate the observed genotype data:

1. Starting at position 0 in *O*, a series of alternating *O* and *M* intervals on the [0,1] scale were generated according to the exponential transition distance distributions for the two-state Markov chain with call rate *λ* over a sequence of length *L*;
2. The genotypes of variants with [0,1] scaled sequence positions not falling within an *O* interval were set to missing.

The observation process defined by this Markov chain is independent of both affection status and underlying genotypes, meaning that missing genotypes are missing completely at random in the sense of Little and Rubin [18].

### Association Testing

For each data set, several methods for association testing were applied. To ensure comparability across data sets from a population, the minor allele was determined based on the allele frequencies in the haplotype population from which the case-control sample was drawn. Variants that were monomorphic in a given data set were excluded from the test. A test producing a p-value rejected the null hypothesis in a data set at level *α* if the p-value was less than or equal to *α*.

Single-variant Cochran-Armitage trend chi-square statistics were calculated as *T* = *N^O^ρ^2^*, where *N^O^* is the number of individuals with observed genotypes at the variant and *ρ* is the Pearson correlation coefficient between the number of minor alleles at the variant and a case status indicator equaling 1 for cases and 0 for controls across individuals with observed genotypes. Locus-wide inference was then performed using these single-variant statistics in accordance with three established methods.

The most widely known method is the BC-CA test presented above, which uses a conservative approximation that does not make efficient use of the single-variant information for joint inference [17]. This test was implemented by rejecting the null hypothesis in a replicate at level *α* if the maximum Cochran-Armitage trend chi-square statistic (max *T*) in the locus was greater than or equal to the Bonferroni-corrected quantile of the asymptotic null 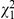 distribution. This Bonferroni-corrected quantile was determined separately for each data set as 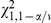, where *v* is the number of polymorphic variants in the data set.

A second popular method involves performing locus-wide inference based on the permutation null distribution of max *T* [19], which is efficient because it does not use a conservative approximation and accounts for the LD-induced correlations between the single-variant *T* values [17]. It has also demonstrated consistently good performance relative to other locus-wide tests in simulations of candidate gene SNPs with realistic LD [19]. The permutation null distribution of max *T* was obtained by repeatedly randomly shuffling affection status labels, calculating all single-variant *T* values, and recording the resulting value of the max *T* statistic. Letting *Q_t_* denote the value of the max *T* statistic in permutation *t* and *Q_obs_* denote the observed value in the sample, the two-sided p-value is estimated from *m* permutations as [35]:
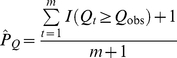
(3)Assuming that missingness does not depend on the underlying genotype or affection status, the Monte Carlo procedure described above will correctly estimate the permutation null distribution and yield a valid p-value. A Monte Carlo estimate of the two-sided p-value was obtained from (3) with *m* = 10,000 permutations. We will refer to the inferential procedure based on the permutation p-value of the max *T* statistic as the CA max test.

A final method involves performing locus-wide inference based on the permutation null distribution of the sum of Cochran-Armitage trend chi-square statistics (sum *T*) over the locus, which is also efficient because LD-induced correlations between single-variant *T* values are fully taken into account in the permutation null distribution. Variations on this theme have been proposed for candidate gene association studies [19] and GWAS [20], [21]. Simulations of candidate gene SNPs with realistic LD found that the approach based on Fisher's method for combining p-values, which is equivalent to a sum of nonnegative single-variant test statistics, performed well relative to other multi-SNP approaches when there were many variants in high LD [19]. A Monte Carlo estimate of the two-sided p-value for the sum *T* statistic was obtained using the same permutation procedure as for the CA max test. We will refer to the inferential procedure based on the permutation p-value of the sum *T* statistic as the CA sum test.

The CA sum test is also closely related to the SKAT and C-alpha test. Let *U_j_* be the score statistic *U* in (1) for a single variant *j* with additive scores 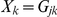, and let 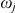 be a pre-specified weight for variant *j*. In the absence of covariates and with complete genotype data, the SKAT statistic can be expressed as 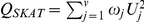 (see Text S1, Appendix C). The authors of the SKAT suggest weights that are a function of a Beta(1,25) density at the pooled sample MAF to increase the contributions of rare variants to the overall sum [13]. With 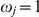 for all *j*, *Q_SKAT_* is equivalent to the C-alpha statistic [13]. With 
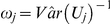
, where 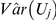 is the estimated null variance of the single-variant Cochran-Armitage trend score statistic from (1), *Q_SKAT_* is equivalent to the sum *T* statistic (see Text S1, Appendix C). With missing genotypes and 
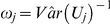
, *Q_SKAT_* remains equivalent to sum *T* when the single-variant SKAT score statistics and 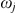 are calculated using all available genotype data at each variant (see Text S1, Appendix C). Thus, the performance of the CA sum test should also provide insight into newer tests that achieve their robustness to neutral and protective variants by performing inference on weighted sums of nonnegative single-variant test statistics.

Our implementation of the CMC method [5] collapsed rare variants having overall sample MAF≤0.01 into an indicator variable equaling 1 if any minor alleles were present and zero otherwise. Common variants that were not collapsed were coded as 0, 1, or 2 minor alleles, and the means of the random vectors comprising the rare variant indicator and common variant minor allele counts were compared between cases and controls using Hotelling's *T* ^2^ test.

One issue not considered in the paper proposing the CMC method is that LD among common variants can induce linear dependency in this random vector, which leads to a singular covariance matrix. However, calculating Hotelling's *T* ^2^ statistic with any generalized inverse is equivalent to calculating the statistic with a standard inverse on a full-rank subset of linearly independent common variants (see Text S1, Appendix D). Goodnight [36] provides an algorithm for automatically calculating a *g2* generalized inverse and the dimension of the full-rank subset without any prior knowledge of the full-rank subset. The algorithm involves applying the G2SWEEP operator once to each of the columns of the covariance matrix in succession. This operator zeros the rows and columns corresponding to common variants that are numerically linearly dependent on the previous common variant minor allele counts and/or the rare variant indicator. The effective number of linearly independent vector elements, *v*, is thus automatically obtained by subtracting the number of columns that are zeroed from the total number of columns in the covariance matrix. The p-value is then calculated using the *F_v_*_,*N-v-*1_ approximation to the distribution of the appropriately scaled Hotelling's *T* ^2^ statistic calculated using the *g2* generalized inverse of the covariance matrix (see Text S1, Appendix D).

Only individuals with complete genotype data at common variants could be used in calculating Hotelling's *T* ^2^. Provided genotype data were complete at all common variants, individuals with missing genotype data at rare variants could be used if at least one minor allele was present for a variant with a non-missing genotype because the coding of the rare variant indicator would be 1 regardless of the other variant genotypes. However, if an individual did not have any minor alleles at any variants with non-missing genotypes, the coding of the rare variant indicator was ambiguous because it would depend on the values of the unobserved genotypes. Therefore, such individuals also had to be excluded from calculating Hotelling's *T* ^2^. With large numbers of exclusions, the *F* test for Hotelling's *T* ^2^ often could not be performed due to insufficient effective denominator degrees of freedom (ddf) or was performed with only a very small number of effective ddf. We considered only results from *F* tests with effective ddf>4 in our Type I error and power estimates because (1) our testing indicated that algebraically identical generalized inverses could yield different numerical results with effective ddf≤4 and (2) the expectation and variance of the *F* distribution only exist for ddf >2 and ddf >4, respectively [27].

Our implementation of the WSS method [6] followed the description in the original paper with four modifications. First, midranks were used to break ties in genetic scores when calculating the case rank-sum statistic, *W*. Second, we used a two-sided p-value. A one-sided p-value will only be well-powered for a deviation from the null in which the cumulative number of minor alleles at lower-frequency variants is higher in cases than controls. However, any departure from the null of equal genotype frequencies in cases and controls at the locus is of interest in association testing, which is why the BC-CA, CA max, CA sum, and CMC tests all use two-sided p-values. Therefore, one would also want to be able to detect deviations in which controls have a higher cumulative number of minor alleles at lower-frequency variants, which is not possible with a one-sided WSS p-value. Such deviations could arise in plausible situations, such as one in which the minor allele of a rare risk variant with a strong effect appears exclusively on a haplotype with few other minor alleles. Third, we estimated the two-sided p-value directly from the permutation distribution of *W*. Letting *W_t_* denote the value in permutation *t*, *W_obs_* denote the observed value in the sample, and 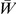
denote the mean of *W* over all *m* permutations, the two-sided p-value was estimated from *m* = 10,000 permutations as [35], [37]:
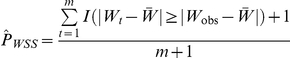
Finally, missing single-variant genotypes, which were not considered in the paper proposing the WSS method, were not used in estimation of the MAF in controls and were assigned values of 0 so as not to contribute to the WSS in an individual. This procedure is equivalent to calculating the genetic score over only nonmissing genotypes in each individual.

## Results

### Characteristics of Variants in Actual Sequence Data

We began by analyzing the MAF and LD distributions of variants in actual sequence data from six candidate genes for dilated cardiomyopathy. These genes spanned a total of 236,059 base pairs (bp), of which 53,466 bp were scanned for variation. A total of 215 biallelic variants were identified in 184 Coriell samples of white European ancestry, yielding approximately 4 variants per kb scanned. We found no evidence against HWE (Monte Carlo exact *P*≥0.001) at 211 of these variants, which were carried forward to the analysis of MAF and LD distributions.

**Figure 1:**
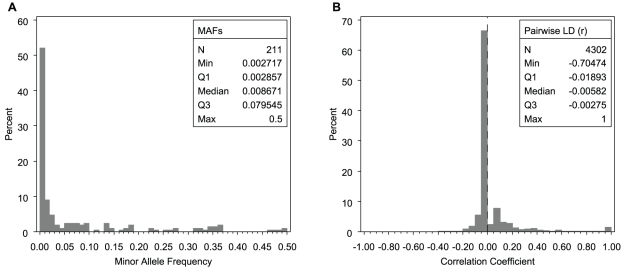
MAF and within-gene pairwise LD distributions in actual sequence data. Distributions of MAFs (Panel A) and within-gene pairwise LD (Panel B) for biallelic variants in six candidate genes for dilated cardiomyopathy. Pairwise LD was measured by the correlation coefficient (*r*) between major/minor alleles for variants within the same gene. These distributions were estimated from 184 Coriell samples of European descent. The vertical dashed line in Panel B indicates *r* = 0.

More than half of variants had MAFs below 0.01, confirming that a multitude of rare variants is likely to be a distinguishing characteristic of sequence data (Figure 1, Panel A). In addition, the majority of pairwise LD between variants within the same gene was small and negative, with more than 75% of *r* values below 0 (Figure 1, Panel B). Pairwise LD between rare variants with MAF≤0.01 was even more concentrated in small negative values, with 95% of values falling between *r* = −0.0095 and *r* = −0.0028. These results confirmed in actual data predictions regarding the sampling distribution of LD based on coalescent theory [14].

Although the negative pairwise *r* values between variants with MAF≤0.01 may seem small in magnitude, they are not inconsistent with negative LD having a substantial impact on pooling tests. First, the theoretical minimum for *r* between variants with MAF≤0.01 is *r*_min_ = −0.0101, so many of these *r* values may actually represent *D′* values near 1 that would be considered strong LD. Second, because neutral variants are far more numerous than risk variants in the genome, an appreciably higher MAF at a single risk variant in cases can mean slightly higher MAFs at numerous neutral variants in controls when most *r* values are negative. If truly neutral variants are not detected with high sensitivity and filtered out prior to analysis, the cumulative case-control MAF difference over this large number of neutral variants can easily mask the cumulative case-control MAF difference over a few risk variants. Therefore, small negative pairwise *r* values between rare variants can have an appreciable effect on pooling tests.

### Impact of Neutral Variation in a Simple Model

**Figure 2:**
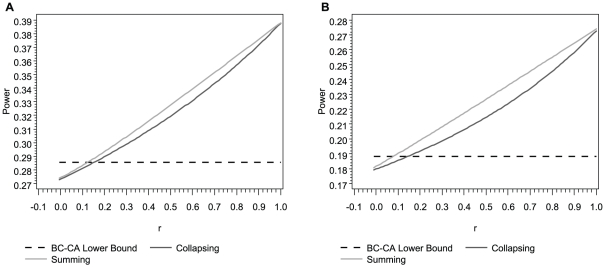
Analytic power comparisons in a small sample (*N* = 500). Analytic locus-wide power at *α* = 0.05 of the BC-CA (lower bound), collapsing, and summing tests at a locus comprising one neutral and one risk variant as a function of the pairwise correlation coefficient between major/minor alleles (*r*). The variants had the same MAF = 0.005 (Panel A) or MAF = 0.01 (Panel B), and the relative risk was 3 (Panel A) or 2 (Panel B) for each additional minor allele at the risk variant. Both panels assume penetrance of 0.05 for the major allele homozygote at the risk variant and a balanced case-control sample with *N* = 500 total subjects.

We used a simple model to compare the power of the BC-CA, collapsing, and summing tests to detect a locus-wide association driven by a single rare risk variant in the presence of a single rare neutral variant at varying levels of LD between the two variants. Although the power of the collapsing and summing tests exceeded the lower bound for the BC-CA test with larger positive *r* values in small samples (*N* = 500), the worst-case power of the BC-CA test was greater than that of the collapsing and summing tests for all *r*≤0.08 under both models considered (Figure 2). Moreover, the BC-CA test had a power advantage over an even larger range of *r* values under the same models in moderate (*r*≤0.34) and large (*r*≤0.52) samples (Figures S1 and S2). In our actual sequence data, over 95% of *r* values between variants with MAFs in the range considered in Figures 2, S1, and S2 (≤0.01) fell below 0, suggesting that the BC-CA test should enjoy a power advantage in most practical situations.

We can explain the relationship between the power of the three tests and *r* by first considering the properties of the BC-CA, collapsing, and summing tests when *r* = 1. In this situation, the number of minor alleles at the neutral variant must always equal the number of minor alleles at the risk variant because minor alleles at both variants must appear on the same haplotype. Thus, the multi-variant genotype frequencies are the same as the genotype frequencies at the risk variant alone, and the scores are *X_k_* = 1, 0, or 0 (collapsing) or *X_k_* = 0, 2, or 4 (summing) when *G_2k_* = 0, 1, or 2, respectively. Under these circumstances, the collapsing and summing tests are equivalent to level *α* single-variant Cochran-Armitage tests for the risk variant using scores for a dominant model and an additive model, respectively. Because these tests are not Bonferroni corrected, their power when *r* = 1 is substantially above the lower bound for the BC-CA test, which is based on a level *α*/2 single-variant Cochran-Armitage test for the risk variant using scores for an additive model.

However, as *r* decreases, the amount of noise introduced into the collapsing and summing test statistics by including the neutral variant increases and results in a concomitant decrease in power. Moreover, the problem of masking by the rare neutral variant further reduces power when *r*<0. The worst-case power of the BC-CA test, which inefficiently combines single-variant test statistics, was substantially greater than the power of the collapsing and summing tests for *r*<0, which is where over 95% of the *r* values between variants with MAF≤0.01 fell in our actual sequence data. These results suggest that, by eliminating the problems of noise and masking, even inefficient techniques for locus-wide inference using nonnegative single-variant test statistics can yield more powerful tests for association than pooling minor alleles in the presence of rare neutral variants.

### Monte Carlo Simulations

Monte Carlo simulations were performed to extend the analytic power results to more realistic situations. These simulations were based on case-control samples generated at a hypothetical 100 kb disease locus with heterogeneous risk alleles, extensive neutral variation, realistic patterns of LD, and randomly missing genotypes.

The variants in our populations of haplotypes simulated based on a coalescent model closely resembled those analyzed in our actual sequence data. First, the rates of variants per kb were compatible when the sampling process that generated our actual sequence data was taken into account. Each simulated population had an average of 981 variant sites (range: 805–1193) over the 100 kb locus, or approximately 10 variants per kb. While this rate was somewhat higher than the observed rate of 4 variants per kb scanned in our actual sequence data, it was not inconsistent with this observation because fewer variants are expected to be observed in any small sample from a large population. In fact, an average of only 747 variants, or 7.5 per kb, appeared in samples of 500 individuals drawn from these haplotype populations under a null disease model with complete genotype data. A further reduction in the number of variants per kb would be expected in a sample of the same size as our actual sequence data, which was about one-third the size of our smallest simulated samples.

Second, the variant MAF and pairwise LD distributions across the populations of simulated haplotypes (Figure S3) closely resembled those across the six candidate genes for dilated cardiomyopathy (Figure 1). The only noticeable difference between the MAF distributions occurred in the lower quantiles because the sample MAF could not fall below 1/368 = 0.002717 in the actual sequence data. The distributions of pairwise LD, measured by the correlation coefficient, were also similar, with a strong resemblance between the histograms and a close correspondence between the quantiles for actual and simulated data. These results suggested that variants in the average simulated haplotype population had similar MAF and LD spectra to variants in the average resequenced dilated cardiomyopathy candidate gene.

We evaluated the performance of the BC-CA, CA max, CA sum, CMC, and WSS tests in samples drawn from these haplotype populations according to different disease models. For each disease model, we considered balanced case-control samples with *N* = 500, *N* = 1,000, and *N* = 2,000; call rates of 100% (complete data), 99.5%, and 95%; and *α* = 0.05 and 0.01.

**Figure 3:**
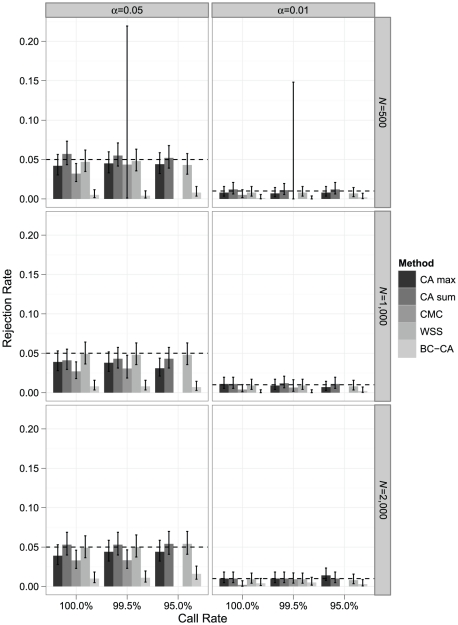
Simulated type I error rate comparison. Monte Carlo estimates of rejection rates for each association testing procedure based on 1,000 samples from a null disease model with no risk variants. Estimates are reported by call rate, nominal *α* level, and sample size (*N*). Error bars represent exact binomial 95% confidence intervals [39] for the rejection rate, and dashed horizontal lines are included at the nominal *α* level. The CMC could not be performed at a call rate of 95% because no individual had complete genotype data in any sample; at a call rate of 99.5%, CMC results with *F* ddf>4 were available in 23, 619, and 992 samples for *N* = 500, 1,000, and 2,000, respectively.

Under a null model with no risk variants, all techniques controlled type I error at the nominal level (Figure 3). The 95% confidence intervals for the CA max, CA sum, and WSS all contained the nominal *α* level under nearly all conditions, reflecting an observed distribution of p-values extremely close to the uniform expected under the null hypothesis. The CMC based on Hotelling's *T* ^2^ was often conservative in complete data, with 95% upper confidence limits below the nominal *α* level. Our result agreed with that of Li and Leal [5], who observed conservatism increasing with the number of variants analyzed when applying Hotelling's *T* ^2^ to a random vector containing between 5 and 20 rare variants in balanced case-control samples of sizes 500 and 2,000. In our null simulations, Hotelling's *T* ^2^ was applied to a much larger vector containing at least 110 effectively linearly independent elements in all data sets with complete genotype data. Finally, the BC-CA test nearly always had the lowest Type I error of all tests considered, reflecting conservatism due to failure to account for LD-induced correlations between single-variant test statistics.

The type I error results for the CMC with missing genotypes also reflect the substantial loss of sample information resulting from having to exclude all individuals with incomplete genotype data at common variants. With a call rate of 95%, no type I error rate could be estimated because no individual had complete data in any of the 1,000 samples and the CMC could not be performed. With a call rate of 99.5%, about 92% of individuals in the average sample were unusable due to missing genotype data for each sample size. For this reason, only 23 of the 1,000 samples had reliable *F* tests with ddf>4 for *N* = 500, and the type I error rate estimates for both *α* levels had wide 95% confidence intervals.

**Figure 4:**
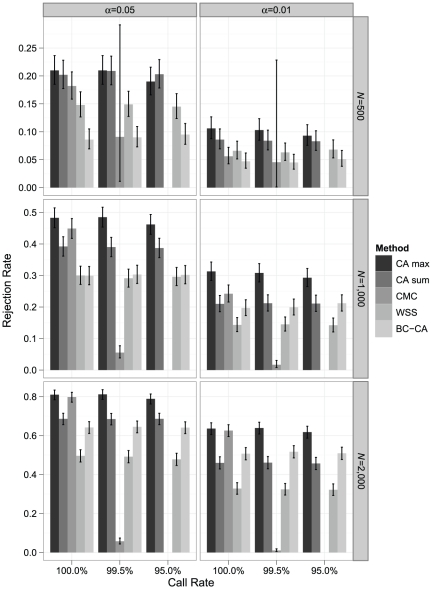
Simulated power comparison for rare risk variants (MAF<0.005; OR = 3). Monte Carlo estimates of rejection rates for each association testing procedure based on 1,000 samples from a disease model with 50 rare risk variants (MAF<0.005; OR = 3), which represent ∼5% of all variants in the locus in the average population. Estimates are reported by call rate, nominal *α* level, and sample size (*N*). Error bars represent exact binomial 95% confidence intervals [39] for the rejection rate. The CMC could not be performed at a call rate of 95% because no individual had complete genotype data in any sample; at a call rate of 99.5%, CMC results with *F* ddf>4 were available in 22, 596, and 991 samples for *N* = 500, 1,000, and 2,000, respectively.

Under a disease model with 50 rare risk variants (MAF<0.005; OR = 3), which represent ∼5% of all variants in the locus in the average haplotype population, the CA max test had higher power than the CMC and WSS tests under all conditions (Figure 4). It also had power comparable to or higher than the CA sum test, which is equivalent to a permutation-based SKAT under an additive genetic model without covariates using the inverse of the estimated null variances of the score statistics as weights. As expected, the CA max test substantially outperformed the BC-CA test, which does not account for LD-induced correlations between test statistics. As the sample size grew, the power of the CMC test with complete data approached that of the CA max test. With missing data, however, the CMC test generally had the lowest power due to the substantial loss of sample when it could even be performed. The CA sum test was more powerful than the CMC test under most conditions, but it began to lag the CMC in complete data for *N*≥1,000. The CA sum test was always more powerful than the WSS test. Although the CA sum test was more powerful the BC-CA test under all scenarios with *α* = 0.05, its power deteriorated to below that of the BC-CA test in larger sample sizes with *α* = 0.01.

Although the WSS test was more powerful than the BC-CA test when *N* = 500, it began to lag the BC-CA test for *N*≥1,000, sometimes substantially. This observation suggests that, when summing over minor alleles to reduce the number of tests performed, the power gain from reducing the multiple-testing penalty was rapidly outweighed by the power loss due to increased noise and masking as the sample size grew. The WSS test also had lower power than the CMC test in most scenarios with complete data. Because the CMC test collapses over only variants with MAF≤0.01 and analyzes common variants in a manner not subject to masking, it may perform better than the WSS test, which sums over all variants.

**Figure 5:**
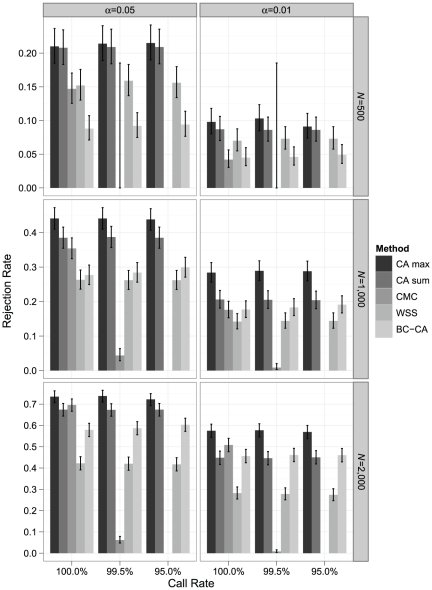
Simulated power comparison for rare risk variants (MAF<0.01; OR = 2). Monte Carlo estimates of rejection rates for each association testing procedure based on 1,000 samples from a disease model with 50 rare risk variants (MAF<0.01; OR = 2), which represent ∼5% of all variants in the locus in the average population. Estimates are reported by call rate, nominal *α* level, and sample size (*N*). Error bars represent exact binomial 95% confidence intervals [39] for the rejection rate. The CMC could not be performed at a call rate of 95% because no individual had complete genotype data in any sample; at a call rate of 99.5%, CMC results with *F* ddf>4 were available in 18, 573, and 986 samples for *N* = 500, 1,000, and 2,000, respectively.

Results were similar under a disease model with 50 rare risk variants (MAF<0.01; OR = 2) (Figure 5). The CA max test had power greater than the CA sum, CMC, WSS, and BC-CA tests under all scenarios. The CA sum test continued to have higher power than the WSS test under all scenarios and was also more powerful than the CMC test for all conditions except *N* = 2,000 with complete data. The CA sum test was more powerful than the BC-CA test under all conditions other than *N* = 2,000 and *α* = 0.01. The WSS test also exhibited a similar pattern of performance relative to the CMC and BC-CA tests.

**Figure 6:**
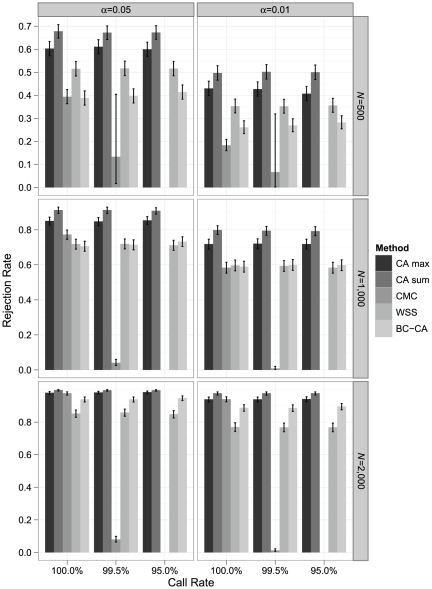
Simulated power comparison for a mixture of rare and common risk variants. Monte Carlo estimates of rejection rates for each association testing procedure based on 1,000 samples from a disease model with 50 total risk variants, which represent ∼5% of all variants in the locus in the average population, randomly allocated between rare variants (MAF<0.01; OR = 2), low-frequency variants (0.01≤MAF<0.05; OR = 1.5), and common variants (0.05≤MAF<0.10; OR = 1.2). Estimates are reported by call rate, nominal *α* level, and sample size (*N*). Error bars represent exact binomial 95% confidence intervals [39] for the rejection rate. The CMC could not be performed at a call rate of 95% because no individual had complete genotype data in any sample; at a call rate of 99.5%, CMC results with *F* ddf>4 were available in 15, 564, and 989 samples for *N* = 500, 1,000, and 2,000, respectively.

In the disease model with 50 total risk variants randomly allocated between rare variants (MAF<0.01; OR = 2), low-frequency variants (0.01≤MAF<0.05; OR = 1.5), and common variants (0.05≤MAF<0.10; OR = 1.2), the CA max and CA sum tests were both more powerful than the CMC and WSS tests under nearly all conditions (Figure 6). Under this disease model, the CA sum test, rather than the CA max test, had the highest power under all conditions. The CA max and CA sum tests also both had greater power than the BC-CA test in all scenarios. With *N* = 2,000 and complete data, the CMC test had comparable power to the CA max test but was still less powerful than the CA sum test. The WSS test showed the same pattern of having higher power than the CMC and BC-CA tests for *N* = 500 but beginning to lag these tests for *N*≥1,000.

## Discussion

We have compared existing methods for efficient locus-wide inference using nonnegative single-variant test statistics to two widely cited pooling tests in terms of their ability to detect associations between rare variants and disease. We began our analysis by exploring the characteristics of variants likely to appear in actual sequence data. Based on these characteristics and a simple model of a locus with one rare risk and one rare neutral variant, we were able to demonstrate that even using Bonferroni-corrected single-variant tests for locus-wide inference may have higher power than collapsing or summing rare variant minor alleles in the presence of a neutral variant. We then simulated populations of haplotypes at a hypothetical 100 kb locus with MAF and LD spectra closely matching those of our actual candidate gene sequence data. We examined power in balanced case-control samples drawn from these simulated haplotype populations according to a disease model with heterogeneous risk alleles and extensive neutral variation. In these simulations, one or more of the existing approaches for efficient locus-wide inference using nonnegative single-variant test statistics, the CA max test or CA sum test, had power comparable to or greater than the CMC and WSS tests under the scenarios considered. Moreover, the type I error and power of the CA max and CA sum tests were robust to randomly missing genotype data, which was not observed with the CMC test. Finally, the CA max test was nearly always more powerful than the CA sum test for disease models with only rare risk variants, suggesting that the CA max test may outperform the class of techniques represented by the SKAT and C-alpha test in these scenarios.

Our results contradict those of the original studies [5], [6] suggesting that the CMC and WSS tests were superior to locus-wide inference using nonnegative single-variant test statistics. However, our simulations improve upon these studies in two important ways that explain the differences in results and make our results more relevant to the analysis of actual sequence data. First, we used a widely accepted population genetic model, the coalescent, to simulate variants with MAF and LD distributions similar to those in actual sequence data, meaning that our simulations should more accurately reflect the impact of neutral variants on each method. Although the CMC study did consider the impact of including neutral variants, it used analytic power calculations that assumed independence between genotypes at different variant sites [5]. The study also considered only models with fixed numbers of variants of different types having equal MAFs within each type. The WSS study considered only MAF spectra consistent with mildly deleterious mutations and sampled each variant, whether risk or neutral, independently of all others [6]. Neither of these methods is likely to recapitulate the rich complexity of the variant MAF and LD distributions that we observed in actual sequence data as well as our coalescent-based approach did. Moreover, simulated data without many higher-frequency neutral variants or substantial LD between neutral and risk variants would tend to cause fewer problems with noise and masking in pooling tests, resulting in overly optimistic assessments of the performance of these techniques. In fact, we found that the WSS test was often less powerful than even the inefficient BC-CA test, suggesting that noise and masking from neutral variants may present major problems for techniques based on summing in actual sequence data.

Second, we used efficient methods for locus-wide inference based on nonnegative single-variant test statistics that reduce the multiple-testing penalty by accounting for LD-induced correlations between the single-variant test statistics. However, the CMC and WSS tests were both compared in the original studies to the Bonferroni and Dunn-Sidak corrections [5], [6], which are both generally conservative. Although the choice to assume independence between variants should mean that the Dunn-Sidak correction was efficient in the original WSS study, the Bonferroni correction used in the original CMC study should still have been conservative and thus inefficient under these conditions. In our more realistic simulated data, LD would have induced correlations between test statistics at different variants, which would have rendered both of these techniques more conservative [17]. In such situations, methods based on simulating the joint distribution of p-values or test statistics under the locus-wide null hypothesis yield more powerful locus-wide tests [17] and are the relevant targets for comparison. The CA max test used in this paper is one such method, and it outperformed the BC-CA test under every scenario considered in our simulations while controlling the Type I error rate, as predicted by theory. Thus, the CA max test, which is simple and computationally feasible, provides a fairer representation of the performance of existing methods for efficient locus-wide inference using nonnegative single-variant test statistics in actual sequence data.

These methods also make use of all available genotype data and are therefore robust to randomly missing genotypes. This robustness stands in stark contrast to our observations for the CMC test using Hotelling's *T* ^2^, which rapidly became unreliable with as little as 0.5% randomly missing genotypes. Other multivariate techniques that rely on a generalized linear model framework, such as the SKAT, will also be subject to the same problem because generalized linear models can only use individuals with complete data. Although all individuals' data could be made complete by imputing missing genotypes, low-frequency or rare variants may be difficult to impute with high accuracy. One caveat to our robustness result is that any method relying on the permutation null distribution for inference, which includes the CA max, CA sum, and WSS tests, will only be valid if genotype missingness does not depend on either affection status or the unknown value of the underlying genotype. In other words, genotypes must be missing completely at random in the sense of Little and Rubin [18]. If this is not the case, affection status is not exchangeable under the genetic null hypothesis, meaning that permutation inference may not yield valid results.

The problem of neutral and protective rare variants masking case-control differences in pooling tests has been recognized by other authors [8]–[13]. Many new developments have therefore sought to reduce the influence of putative neutral and protective variants using filtering, classification, or weights based on annotation, functional predictions, or MAFs [8]–[11], [13]. While these approaches seem sensible, there are several drawbacks. First, annotation and functional predictions are not readily available for non-coding sequences that may influence disease through recently discovered or as-yet-unknown regulatory mechanisms. Second, as demonstrated by recent examples implicating synonymous coding variants in altered protein products and Crohn's disease [15], [16], annotation and functional predictions for coding sequences do not always provide a solid basis on which to separate putative risk, neutral, and protective variants a priori. Finally, distinguishing neutral and protective variants based on sample MAFs alone [11] will be prone to error because of sampling variability, particularly with rare variants. In contrast, methods for locus-wide inference using nonnegative single-variant test statistics are inherently robust to the inclusion of neutral and protective variants and may even be able to exploit their LD with risk variants to increase power. Notably, the power advantage of the CA max and CA sum tests observed in this study did not require any information or assumptions about the putative functional consequences of the minor allele in relation to the disease of interest. Thus, the CA max or CA sum tests could be applied equally well to coding sequence, non-coding sequence with poorly understood functional consequences, or a combination thereof.

An additional advantage of applying existing methods for locus-wide inference using nonnegative single-variant test statistics is their adaptability. Although we have focused on single-locus inference for concreteness, test statistics can be combined over any relevant grouping of variants, including single exons, pathways, or the entire exome, to perform joint inference. Pooling tests can also be applied to arbitrary groupings, but they are not inherently robust to the inclusion of neutral and protective variants. Moreover, although we focused on case-control association testing in the absence of confounding and population stratification, existing methods using nonnegative single-variant test statistics can be readily extended to multi-variant joint inference in more complex case-control or family-based designs by simply changing the test statistic and permutation strategy. As long as the new test statistic has a nonnegative value that depends only on the magnitude of the deviation from the statistical null hypothesis at each variant, the locus-wide test is inherently robust to the inclusion of neutral and protective variants. The permutation strategy would then need to be adapted to ensure exchangeability under the model implied by the new single-variant test statistic (see, e.g., McIntyre et al. [38] for a permutation strategy valid for the transmission/disequilibrium test statistic in a trio design). Finally, although we considered only the maximum and sum of Cochran-Armitage trend chi-square statistics over the variant grouping of interest, almost any summary of a wide variety of nonnegative single-variant test statistics could be used for joint inference based on the appropriate permutation distribution.

Although the idea of pooling minor alleles in association tests with rare variants may still hold sway in the genetics community, it is worth noting that some new association tests with greater robustness to the inclusion of neutral and protective variants have implicitly returned to locus-wide inference using nonnegative single-variant test statistics. Specifically, the SKAT [13] and C-alpha test [12] are equivalent to basing inference on weighted and unweighted sums of squared single-variant score statistics, respectively [13]. The sum *T* statistic evaluated in this study is also a sum of squared single-variant score statistics weighted by the inverse of their estimated null variances. Our results for the CA sum test, combined with the results of the studies proposing the SKAT and C-alpha test [12], [13], suggest that further extending methods for locus-wide inference using nonnegative single-variant test statistics may be a fruitful line of research. Moreover, a method in this class fundamentally different from the closely related SKAT, C-alpha test, and CA sum test—the CA max test—often had greater power than the CA sum test for disease models with only rare risk variants. We therefore suggest that a conceptual framework based on optimally combining nonnegative single-variant test statistics may yield useful insights or suggest other existing techniques that might be overlooked within a conceptual framework based on pooling minor alleles.

## Supporting Information

Figure S1 **Analytic power comparisons in a moderate sample (** ***N*** **= 1,000).** Analytic locus-wide power at *α* = 0.05 of the BC-CA (lower bound), collapsing, and summing tests at a locus comprising one neutral and one risk variant as a function of the pairwise correlation coefficient between major/minor alleles (*r*). The variants had the same MAF = 0.005 (Panel A) or MAF = 0.01 (Panel B), and the relative risk was 3 (Panel A) or 2 (Panel B) for each additional minor allele at the risk variant. Both panels assume penetrance of 0.05 for the major allele homozygote at the risk variant and a balanced case-control sample with *N* = 1,000 total subjects.
(PNG)

Figure S2 **Analytic power comparisons in a large sample (** ***N*** **= 2,000).** Analytic locus-wide power at *α* = 0.05 of the BC-CA (lower bound), collapsing, and summing tests at a locus comprising one neutral and one risk variant as a function of the pairwise correlation coefficient between major/minor alleles (*r*). The variants had the same MAF = 0.005 (Panel A) or MAF = 0.01 (Panel B), and the relative risk was 3 (Panel A) or 2 (Panel B) for each additional minor allele at the risk variant. Both panels assume penetrance of 0.05 for the major allele homozygote at the risk variant and a balanced case-control sample with *N* = 2,000 total subjects.
(PNG)

Figure S3 **MAF and pairwise LD distributions in simulated sequence data.** Distributions of MAFs (Panel A) and pairwise LD (Panel B) for biallelic variants in 1,000 populations of 10,000 simulated haplotypes each at a 100 kb locus. Pairwise LD was measured by the within-gene pairwise correlation coefficient (*r*) between major/minor alleles. Because it was computationally infeasible to summarize hundreds of millions of pairwise LD values, a 0.1% simple random sample of these values was taken from each haplotype population. We repeated this sampling procedure several times and obtained similar results. The vertical dashed line in Panel B indicates *r* = 0.
(PNG)

Text S1 **Appendices.**
(DOC)
