# Supplementary figures and images for: Reconsidering Association Testing Methods Using Single-Variant Test Statistics as Alternatives to Pooling Tests for Sequence Data with Rare Variants
Source: PLoS One. 2012 Feb 17;7(2):e30238. doi: 10.1371/journal.pone.0030238 (PMC3281828; doi:10.1371/journal.pone.0030238)

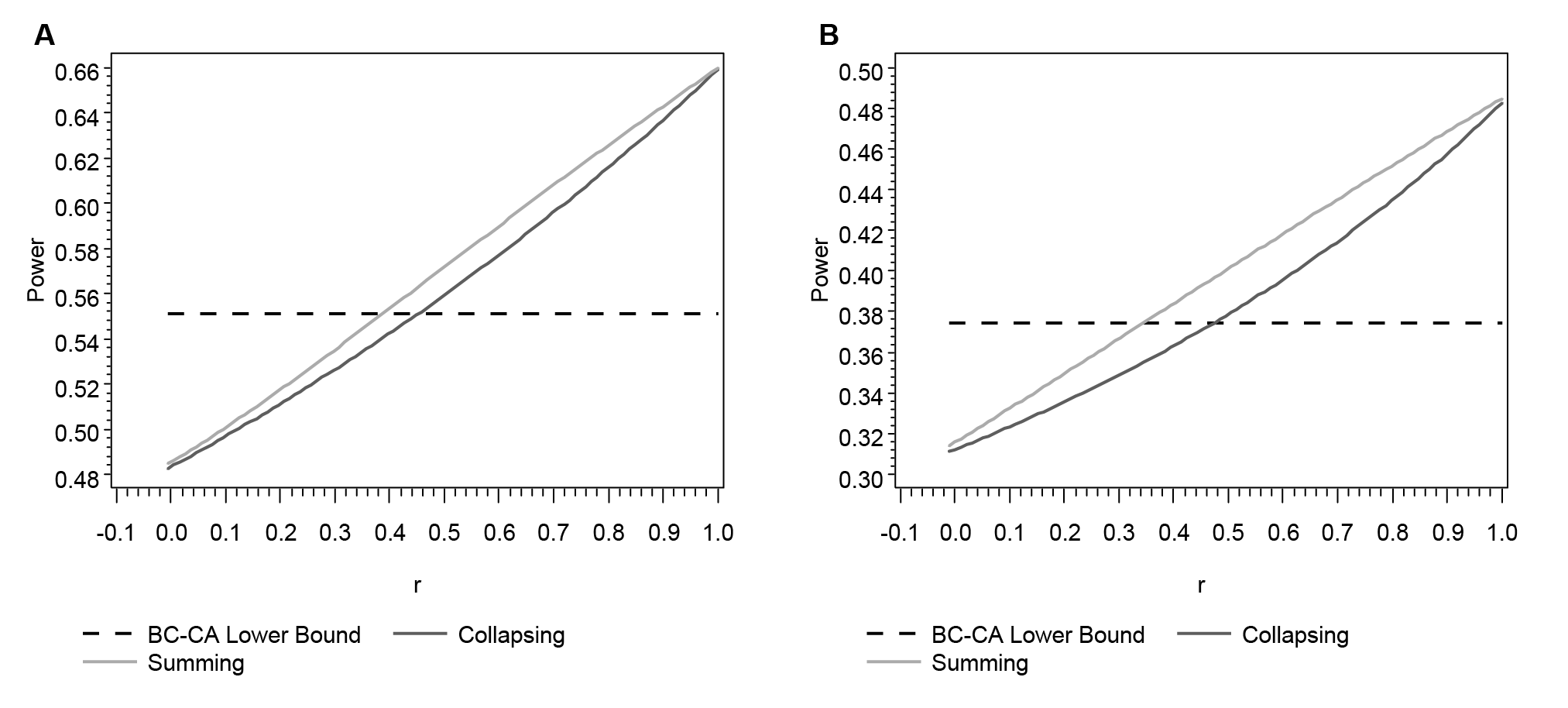

Supplement: Figure S1 — Analytic power comparisons in a moderate sample ( N = 1,000). Analytic locus-wide power at α = 0.05 of the BC-CA (lower bound), collapsing, and summing tests at a locus comprising one neutral and one risk variant as a function of the pairwise correlation coefficient between major/minor alleles (r). The variants had the same MAF = 0.005 (Panel A) or MAF = 0.01 (Panel B), and the relative risk was 3 (Panel A) or 2 (Panel B) for each additional minor allele at the risk variant. Both panels assume penetrance of 0.05 for the major allele homozygote at the risk variant and a balanced case-control sample with N = 1,000 total subjects. (PNG) [file pone.0030238.s001.png]

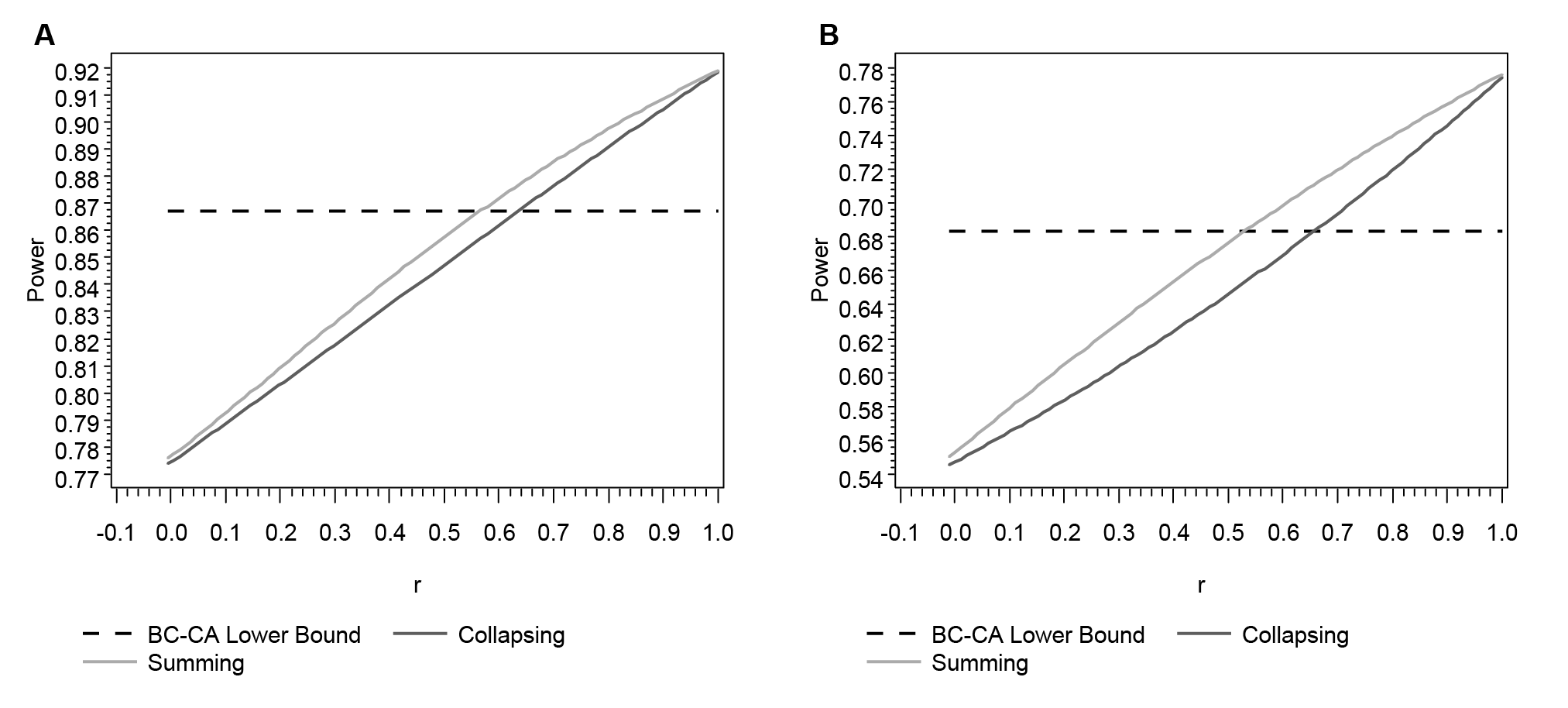

Supplement: Figure S2 — Analytic power comparisons in a large sample ( N = 2,000). Analytic locus-wide power at α = 0.05 of the BC-CA (lower bound), collapsing, and summing tests at a locus comprising one neutral and one risk variant as a function of the pairwise correlation coefficient between major/minor alleles (r). The variants had the same MAF = 0.005 (Panel A) or MAF = 0.01 (Panel B), and the relative risk was 3 (Panel A) or 2 (Panel B) for each additional minor allele at the risk variant. Both panels assume penetrance of 0.05 for the major allele homozygote at the risk variant and a balanced case-control sample with N = 2,000 total subjects. (PNG) [file pone.0030238.s002.png]

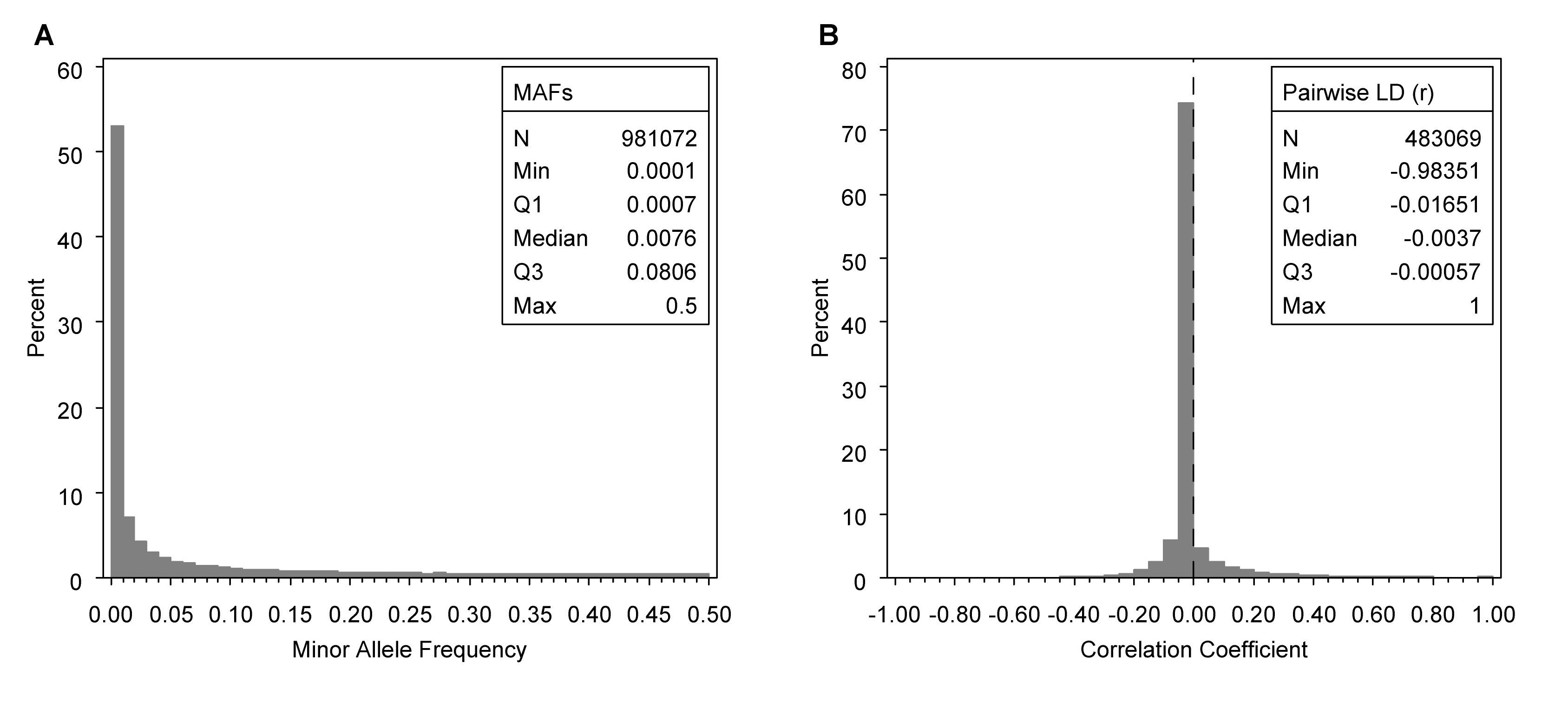

Supplement: Figure S3 — MAF and pairwise LD distributions in simulated sequence data. Distributions of MAFs (Panel A) and pairwise LD (Panel B) for biallelic variants in 1,000 populations of 10,000 simulated haplotypes each at a 100 kb locus. Pairwise LD was measured by the within-gene pairwise correlation coefficient (r) between major/minor alleles. Because it was computationally infeasible to summarize hundreds of millions of pairwise LD values, a 0.1% simple random sample of these values was taken from each haplotype population. We repeated this sampling procedure several times and obtained similar results. The vertical dashed line in Panel B indicates r = 0. (PNG) [file pone.0030238.s003.png]
