# Supplementary material for: Reconsidering Association Testing Methods Using Single-Variant Test Statistics as Alternatives to Pooling Tests for Sequence Data with Rare Variants
Source: PLoS One. 2012 Feb 17;7(2):e30238. doi: 10.1371/journal.pone.0030238 (PMC3281828; doi:10.1371/journal.pone.0030238)
Supplement: Text S1 — Appendices. (DOC) [file pone.0030238.s004.doc]

**S1. Appendices**

**Appendix A**

We prove that particular choices of scores in the generalized Cochran-Armitage test statistic defined by equation (1) in the main text yield a single-variant Cochran-Armitage trend test, a Pearson chi-square test for independence between the presence/absence of minor alleles at the locus and case status (collapsing), or a *Z* test for differences in the mean minor allele count between cases and controls (summing). For the single-variant Cochran-Armitage trend test, let *X_k_=G_jk_*, *r_l_* be the count of cases with *G_jk_*=*l* (*l*=0, 1, or 2), *s_l_* be the count of controls with *G_jk_*=*l*, and *n_l_*=*r_l_*+*s_l_*. The formulas for *U* and its estimated null variance in (1) can be expressed as:

These expressions are identical to those for the single-variant Cochran-Armitage trend test in Freidlin et al. [1]. The square of the test statistic presented in this reference has the same asymptotic$\chi_{1}^{2}$ null distribution as the generalized Cochran-Armitage chi-square statistic defined in (1).

For the collapsing test, we could use the Pearson chi-square test for independence between the presence/absence of minor alleles at the locus and case status. Without loss of generality, let **G_1_** be the multi-variant genotype where *G_j_*_1_ = 0 for all *j* (i.e., that contains no minor alleles),$X_{k}=1$ for *k*=1, and $X_{k}=0$ for *k*>1. Then:

where $r_{\neg1}$ and $s_{\neg1}$ denote the numbers of cases and controls, respectively, with genotypes other than **G_1_**. Thus, *T* with this choice of scores exactly equals the desired Pearson chi-square statistic [2], which has the same asymptotic$\chi_{1}^{2}$ null distribution as the generalized Cochran-Armitage chi-square statistic defined in (1).

For the summing test, we could compare the mean minor allele count over the locus between cases and controls using a *Z* test. Suppose that we choose $X_{k}=\sum_{j} G_{jk}$, the number of minor alleles summed across variants in the multi-variant genotype **G_k_**, as scores. Then:

Thus, *T* is the square of a *Z* statistic for the difference in mean minor allele counts between cases and controls using a variance estimate that is consistent under the null hypothesis. In large samples, this *Z* statistic has a standard normal distribution under the null hypothesis, meaning that its square has the same asymptotic$\chi_{1}^{2}$ null distribution as the generalized Cochran-Armitage chi-square statistic defined in (1).

**Appendix B**

We establish that a lower bound for the locus-wide power function of the BC-CA test in our model of a two-variant locus is the power of the Bonferroni-corrected Cochran-Armitage trend test for the risk variant alone. The BC-CA test will reject the null for the locus if the maximum test statistic in the locus exceeds the Bonferroni-corrected critical value. Thus, rejection by the BC-CA test comprises 3 disjoint events:

1. The Cochran-Armitage trend chi-square statistic for variant 1 (neutral) is greater than the Bonferroni-corrected critical value ($T_{1}>\chi_{1,1-\alpha/2}^{2}$), and the Cochran-Armitage trend chi-square statistic for variant 2 (risk) is not ($T_{2}\leq\chi_{1,1-\alpha/2}^{2}$);
2. $T_{1}\leq\chi_{1,1-\alpha/2}^{2}$and $T_{2}>\chi_{1,1-\alpha/2}^{2}$;
3. $T_{1}>\chi_{1,1-\alpha/2}^{2}$and $T_{2}>\chi_{1,1-\alpha/2}^{2}$

Thus, the locus-wide power function corresponds to the probabilities of these 3 disjoint events under the alternative hypothesis:

where the second equality follows from the fact that summing over both possible events with respect to neutral variant 1 in the second and third terms yields the marginal probability of a rejection at the risk variant 2. The first term is quite difficult to evaluate without assumptions regarding the joint distribution of the single-variant test statistics, but it is always greater than or equal to zero because it is a probability. Thus, in the presence of LD:

**Appendix C**

We first show that, in the absence of covariates and with complete genotype data, the SKAT statistic can be expressed as $Q_{SKAT}=\sum_{j=1}^{v} \omega_{j}U_{j}^{2}$, where *U_j_* is the score statistic *U* in (1) for a single variant *j* with scores $X_{k}=G_{jk}$ and$\omega_{j}$ is a pre-specified weight for variant *j*. Following the development in Wu et al. [3], let *y_i_* be a 1/0 case/control indicator variable and *G_ij_* be the number of minor alleles (0, 1, or 2) for individual *i* at variant *j*. Consider a logistic regression model for a single variant in a case-control study without additional covariates:

$$\text{logit }P\left(y_{i}=1\right)=\alpha_{0}+\beta_{j}G_{ij}$$

The SKAT statistic can be expressed as $Q_{SKAT}=\sum_{j=1}^{v} \omega_{j}U_{j}^{2}$, where is the single-variant score statistic for testing the null hypothesis that $\beta_{j}=0$ for variant *j* and for all *j* [3]. If we let $\mathbf{G}_{\mathbf{k}}=\left[G_{jk}\right]$, *k*=1, 2, or 3, *U_j_* can also be expressed as:

$$\begin{matrix} U_{j}=\mathbf{G}_{\mathbf{j}}^{\text{T}}\left(\mathbf{y}-\frac{R}{N}\mathbf{1}_{N\times1}\right)=\sum_{i=1}^{N} G_{ij}\left(y_{i}-\frac{R}{N}\right) \\ =\sum_{i=1}^{N} \sum_{k=1}^{3} I\left(G_{ij}=G_{jk}\right)\left[G_{jk}\left(y_{i}-\frac{R}{N}\right)\right] \\ =\sum_{k=1}^{3} G_{jk}\left(\sum_{i=1}^{N} I\left(G_{ij}=G_{jk}\right)y_{i}-\sum_{i=1}^{N} I\left(G_{ij}=G_{jk}\right)\frac{R}{N}\right) \\ =\sum_{k=1}^{3} G_{jk}\left(r_{k}-\frac{R}{N}n{}_{k}\right)=\sum_{k=1}^{3} G_{jk}\left(\frac{R+S}{N}r_{k}-\frac{R}{N}\left(r_{k}+s{}_{k}\right)\right) \\ =\sum_{k=1}^{3} G_{jk}\left(\frac{S}{N}r_{k}-\frac{R}{N}s{}_{k}\right) \end{matrix}$$

Thus, *U_j_* is identical to *U* in (1) of the main text with $\mathbf{G}_{\mathbf{k}}=\left[G_{jk}\right]$, *k*=1, 2, or 3, and$X_{k}=G_{jk}$, which completes the proof.

We now demonstrate that, with $\omega_{j}=V\hat{a}r\left(U_{j}\right)^{-1}$, where $V\hat{a}r\left(U_{j}\right)$ is the estimated null variance of the single-variant Cochran-Armitage trend score statistic from (1) of the main text, *Q_SKAT_* is equivalent to the sum *T* statistic. This choice of weights increases the contributions of rare variants to the overall SKAT statistic in a principled manner by weighting sum components so that they have the same marginal asymptotic distribution under the null hypothesis. With these weights, we have:

*T_j_* is clearly the single-variant Cochran-Armitage trend chi-square statistic for variant *j* from (1) of the main text, which completes the proof.

With missing genotypes and $\omega_{j}=V\hat{a}r\left(U_{j}\right)^{-1}$, we show that *Q_SKAT_* remains equivalent to sum *T* when the single-variant SKAT score statistics and $\omega_{j}$ are calculated using all available genotype data at each variant. For an arbitrary variant *j*, let $\mathbf{O}_{\mathbf{j}}$ be a vector of length *N* containing 1 for individuals with available genotype data and 0 otherwise. Also let a superscript *O* on a previously defined quantity denote that it was calculated in all individuals with available genotype data at variant *j*. The single-variant score statistic for testing the null hypothesis that $\beta_{j}=0$ in the $N_{j}^{O}$ individuals with available genotype data at variant *j* is , where . $U_{j}^{O}$ can also be expressed as:

$$\begin{matrix} U_{j}^{O}=\left(\mathbf{O}_{\mathbf{j}}\circ\mathbf{G}_{\mathbf{j}}\right)^{\text{T}}\left(\mathbf{y}-\frac{R_{j}^{O}}{N_{j}^{O}}\mathbf{1}_{N\times1}\right)=\sum_{i=1}^{N} O_{ij}G_{ij}\left(y_{i}-\frac{R_{j}^{O}}{N_{j}^{O}}\right) \\ =\sum_{i=1}^{N} \sum_{k=1}^{3} O_{ij}I\left(G_{ij}=G_{jk}\right)\left[G_{jk}\left(y_{i}-\frac{R_{j}^{O}}{N_{j}^{O}}\right)\right] \\ =\sum_{k=1}^{3} G_{jk}\left(\sum_{i=1}^{N} O_{ij}I\left(G_{ij}=G_{jk}\right)y_{i}-\sum_{i=1}^{N} O_{ij}I\left(G_{ij}=G_{jk}\right)\frac{R_{j}^{O}}{N_{j}^{O}}\right) \\ =\sum_{k=1}^{3} G_{jk}\left(r_{k}^{O}-\frac{R_{j}^{O}}{N_{j}^{O}}n_{k}^{O}\right)=\sum_{k=1}^{3} G_{jk}\left(\frac{R_{j}^{O}+S_{j}^{O}}{N_{j}^{O}}r_{k}^{O}-\frac{R_{j}^{O}}{N_{j}^{O}}\left(r_{k}^{O}+s_{k}^{O}\right)\right) \\ =\sum_{k=1}^{3} G_{jk}\left(\frac{S_{j}^{O}}{N_{j}^{O}}r_{k}^{O}-\frac{R_{j}^{O}}{N_{j}^{O}}s_{k}^{O}\right) \end{matrix}$$

$U_{j}^{O}$ is therefore identical to *U* in (1) of the main text calculated in the $N_{j}^{O}$ individuals with available genotype data at variant *j* using $\mathbf{G}_{\mathbf{k}}=\left[G_{jk}\right]$, *k*=1, 2, or 3, and$X_{k}=G_{jk}$*.* If we denote $V\hat{a}r\left(U_{j}\right)$ calculated in these individuals by $V\hat{a}r\left(U_{j}^{O}\right)$ and set $\omega_{j}=V\hat{a}r(U_{j}^{O})^{-1}$, we have:

$T_{j}^{O}$ is the single-variant Cochran-Armitage trend chi-square statistic calculated using all available genotypes at variant *j*, which completes the proof.

**Appendix D**

We show that performing Hotelling’s *T*^2^ test on a vector with linearly dependent elements using any generalized inverse is equivalent to selecting a maximal linearly independent subset of *v* of these elements and performing the test in the standard manner on a vector containing only this subset. Let **x_i_** and **y_j_** be independent column vectors of dimension *u* indexed by *i*=1,…,*n_x_* and *j*=1,…,*n_y_*. In what follows, any quantity with a bar over it denotes the mean over *i* or *j*, as appropriate, and a superscript T indicates a transpose. We can write the pooled covariance matrix **S** as:

Hotelling’s *T*^2^ test statistic using a generalized inverse of **S**, $\mathbf{S}^{-}$, is defined as:

(C1)

Suppose that some elements of the **x_i_** and **y_j_** vectors are perfectly predicted by linear combinations of the others, such as if genotypes at a particular variant can be perfectly predicted based on linear combinations of the genotypes at other variants due to LD. In that case, we can find a permutation matrix **P** that rearranges the **x_i_** and **y_j_** vectors such that the first *v* elements contain all the linearly independent elements of the original vectors and the remaining *u*-*v* elements contain all the linearly dependent elements. Mathematically,

$$\begin{matrix} \mathbf{Px}_{\mathbf{i}}=\left[\begin{matrix} \mathbf{x}_{\mathbf{Ii}} \\ \mathbf{x}_{\mathbf{Di}} \end{matrix}\right]=\left[\begin{matrix} \mathbf{x}_{\mathbf{Ii}} \\ \mathbf{Lx}_{\mathbf{Ii}} \end{matrix}\right] \\ \mathbf{Py}_{\mathbf{j}}=\left[\begin{matrix} \mathbf{y}_{\mathbf{Ij}} \\ \mathbf{y}_{\mathbf{Dj}} \end{matrix}\right]=\left[\begin{matrix} \mathbf{y}_{\mathbf{Ij}} \\ \mathbf{Ly}_{\mathbf{Ij}} \end{matrix}\right] \end{matrix}$$

where the subscript **I** refers to the *v* linearly independent elements, the subscript **D** refers to the *u* – *v* linearly dependent elements, and **L** is an arbitrary $\left(u-v\right)\times v$ linear transformation defining the linear dependency of the **D** elements on the **I** elements. If we apply this permutation and its transpose to the covariance matrix, we obtain:

where **S_I_** is the $v\times v$ covariance matrix for the **I** elements.

The application of the permutation matrix **P** to the covariance matrix **S** reorders the rows (left multiplication) and columns (right multiplication) in the same manner. We are interested in where the linearly independent rows and columns of **S** are moved to in **PSP**^T^. Based on the symmetry of **S** and **PSP**^T^, it is evident that the results that hold for the columns of **S** and **PSP**^T^ will hold identically for the rows, so we will proceed to determine the location of only the linearly independent columns of **S** in **PSP**^T^.

We can prove that the first *v* columns of **PSP**^T^, which constitute the submatrix **S_V_**, contain all the linearly independent columns of **S** and only these columns. To show that **S_V_** contains all the linearly independent columns of **S**, it is sufficient to prove that **S_U_** cannot contain any linearly independent column of **S**. By definition, **S_U_=S_V_L**^T^, meaning that all columns of **S** moved to **S_U_** are linear combinations of other columns of **S** moved to **S_V_**. Therefore, **S_U_** cannot contain any linearly independent column of **S**.

We have established that **S_V_** contains all linearly independent columns of **S**. To demonstrate that it contains only the linearly independent columns in **S**, it is sufficient to prove that **S_V_** cannot contain any linearly dependent column of **S**. We begin by noting that, if **S_I_** has full rank, all of its columns must be linearly independent. To show that **S_I_** has full rank, let **X_IC_** be the $n_{x}\times v$ matrix obtained by stacking in the rows. This matrix has rank *v* because the columns corresponding to the vector elements are linearly independent by definition. We can then write the sum of squares and cross products matrix for **x_Ii_** as:

Because $\mathbf{I}_{n_{x}\times n_{x}}$is positive definite and **X_IC_** has rank *v*, $\mathbf{X}_{\mathbf{IC}}^{\text{T}}\mathbf{I}_{n_{x}\times n_{x}}\mathbf{X}_{\mathbf{IC}}$ is also positive definite (Theorem 14.2.9 of Harville [4]). The same reasoning follows for the sum of squares and cross products matrix for **y_Ii_**. Now, **S_I_** can be written as the sum of two positive definite matrices:

$$\mathbf{S}_{\mathbf{I}}=\left(n_{x}+n_{y}-2\right)^{-1}\left[\mathbf{X}_{\mathbf{IC}}^{\text{T}}\mathbf{I}_{n_{x}\times n_{x}}\mathbf{X}_{\mathbf{IC}}+\mathbf{Y}_{\mathbf{IC}}^{\text{T}}\mathbf{I}_{n_{y}\times n_{y}}\mathbf{Y}_{\mathbf{IC}}\right]$$

Because the sum of two positive definite matrices is also positive definite (Lemma 14.2.4 of Harville [4]) and any positive definite matrix is nonsingular (Lemma 14.2.8 of Harville [4]), **S_I_** has full rank. Because **S_V_** contains all the linearly independent columns of **S**, any linearly dependent columns of **S** would be a linear combination of columns moved to **S_V_**. Therefore, if a linearly dependent column in **S** had been moved to **S_V_**, it would be linearly dependent on the other columns of **S_V_**, which would contradict the fact that **S_I_** has full rank. Thus, **S_V_** cannot contain any linearly dependent column of **S**, meaning that **S_V_** contains only linearly independent columns of **S**. This completes the proof that the first *v* columns of **PSP**^T^ contain all the linearly independent columns of **S** and only these columns, and the same result for the first *v* rows of **PSP**^T^ immediately follows from symmetry.

We now have a $u\times u$symmetric matrix **S** of rank *v* < *u* and a permutation matrix **P** that can generate a partitioned matrix **PSP**^T^ in which the first *v* columns and rows are the linearly independent columns and rows of **S**. According to Theorem 9.2.5 of Harville [4], $\mathbf{S}^{-}$ is a generalized inverse of **S** if and only if:

$$\begin{matrix} \mathbf{S}^{-}=\mathbf{P}^{\text{T}}\left[\begin{matrix} \mathbf{S}_{\mathbf{I}}^{-\mathbf{1}}-\mathbf{ALS}_{\mathbf{I}}\mathbf{S}_{\mathbf{I}}^{-\mathbf{1}}-\mathbf{S}_{\mathbf{I}}^{-\mathbf{1}}\mathbf{S}_{\mathbf{I}}\mathbf{L}^{\mathbf{T}}\mathbf{B}-\mathbf{S}_{\mathbf{I}}^{-\mathbf{1}}\mathbf{S}_{\mathbf{I}}\mathbf{L}^{\mathbf{T}}\mathbf{CLS}_{\mathbf{I}}\mathbf{S}_{\mathbf{I}}^{-\mathbf{1}} & \mathbf{A} \\ \mathbf{B} & \mathbf{C} \end{matrix}\right]\mathbf{P} \\ =\mathbf{P}^{\text{T}}\left[\begin{matrix} \mathbf{S}_{\mathbf{I}}^{-\mathbf{1}}-\mathbf{AL}-\mathbf{L}^{\mathbf{T}}\mathbf{B}-\mathbf{L}^{\mathbf{T}}\mathbf{CL} & \mathbf{A} \\ \mathbf{B} & \mathbf{C} \end{matrix}\right]\mathbf{P} \end{matrix}$$

for arbitrary matrices **A**, **B**, and **C** of the appropriate dimensions. Specific types of generalized inverses, such as *g2* and Moore-Penrose generalized inverses, correspond to specific choices of **A**, **B**, and **C** that satisfy the additional conditions required for these types of generalized inverses.

If we substitute the above expression for the generalized inverse into the Hotelling’s *T*^2^ test statistic defined in appendix equation (C1), we obtain:

Thus, using any type of generalized inverse in Hotelling’s *T*^2^ statistic is algebraically equivalent to calculating the statistic using the maximal linearly independent subset of *v* vector elements. However, procedures to calculate the generalized inverse do not require any prior knowledge of which vector elements form this subset, meaning that knowledge of **P** is not required to apply the procedure. Based on the above equivalence, inference on the test statistic defined in (C1) can be performed using the *F* approximation [5]:

$$\frac{n_{x}+n_{y}-v-1}{v}\frac{T^{2}}{n_{x}+n_{y}-2}\sim^{a} F_{v,n_{x}+n_{y}-v-1}$$

These algebraic equivalences may not hold numerically when there are only a few more vectors in the sample than the number of linearly independent elements (i.e., $n_{x}+n_{y}-v-1$ is in the single digits). In such situations, small differences in the numerical algorithms used to calculate each type of generalized inverse may lead to different test statistics due to different roundoff error properties or selection of different maximal linearly independent subsets of vector elements.

**References**

1. Freidlin B, Zheng G, Li Z, Gastwirth JL (2002) Trend tests for case-control studies of genetic markers: power, sample size and robustness. Hum Hered 53: 146-152.

2. Sokal RR, Rohlf FJ (1995) Biometry. 3rd ed. New York: W.H. Freeman.

3. Wu MC, Lee S, Cai T, Li Y, Boehnke M, et al. (2011) Rare-variant association testing for sequencing data with the sequence kernel association test. Am J Hum Genet 89: 82-93.

4. Harville DA (1997) Matrix Algebra from a Statistician’s Perspective. New York: Springer.

5. Timm NH (2002) Applied Multivariate Analysis. New York: Springer.
